# The Optimization of Mechanical Alloying Conditions of Powder for the Preparation of a Fe-10Al-4Cr-4Y_2_O_3_ ODS Nanocomposite

**DOI:** 10.3390/ma15249034

**Published:** 2022-12-17

**Authors:** Jiří Svoboda, Štepán Gamanov, Denisa Bártková, Natália Luptáková, Petr Bořil, Milan Jarý, Bohuslav Mašek, Jakub Holzer, Petr Dymáček

**Affiliations:** 1 Institute of Physics of Materials, Czech Academy of Sciences, Žižkova 22, 616 62 Brno, Czech Republic; 2 CEITEC, Brno University of Technology, Purkyňova 656/123, 612 00 Brno, Czech Republic; 3 Faculty of Electrical Engineering, University of West Bohemia, Univerzitni 8, 306 14 Pilsen, Czech Republic

**Keywords:** oxide dispersion strengthened (ODS) alloy, mechanical alloying (MA), powder hot consolidation by rolling, strength at 1100 °C, nanocomposite

## Abstract

Mechanical alloying (MA) of powders represents the first processing step in the production of oxide dispersion-strengthened (ODS) alloys. MA is a time and energy-consuming process also in the production of Fe-10Al-4Cr-4Y_2_O_3_ creep and oxidation-resistant ODS nanocomposite, denoted as the FeAlOY, and it deserves to be optimized. MA is performed at two different temperatures at different times. The powder after MA, as well as the microstructure and high-temperature strength of the final FeAlOY, are characterized and the optimal MA conditions are evaluated. The obtained results show that the size distribution of the powder particles, as well as the dissolution and homogenization of the Y_2_O_3_, becomes saturated quite soon, while the homogenization of the metallic components, such as Al and Cr, takes significantly more time. The high-temperature tensile tests and grain microstructures of the secondary recrystallized FeAlOY, however, indicate that the homogenization of the metallic components during MA does not influence the quality of the FeAlOY, as the matrix of the FeAlOY is sufficiently homogenized during recrystallization. Thus, the conditions of MA correspond to sufficient dissolution and homogenization of Y_2_O_3_ and can be considered the optimal ones.

## 1. Introduction

An effective solution to climate disruption, as well as planet protection, and the approach of the global society to sustainable development are the central tasks of mankind. Therefore, also a further increase in the efficiency of heat machines by increasing their operating temperatures is of high importance. This sector needs new creep and oxidation-resistant materials allowing for a long-term operation at very high temperatures. These requirements can be met by strengthening metallic materials by dispersion of fine particles as precipitates [1,2,3,4]. Most alloys, such as steels, are strengthened by carbides, nitrides, and carbonitrides [5,6,7]. Their stability is, however, limited by the temperature (about 800 °C) of ferrite to austenite phase transformation, as the precipitates easily dissolve in the austenite. The applications up to 1000 °C can be met by austenitic nickel-based superalloys strengthened by ordered *γ’* precipitates [8]. In the cases where the application temperature reaches a value up to 1300 °C, the strengthening by very stable nanooxides seems to be the only possible solution to the problem. Then, oxide dispersion strengthened (ODS) alloys can be applied, where nanoparticles of oxides, e.g., the most stable Y_2_O_3_, are homogeneously dispersed in the matrix [9,10].

The ODS alloys are investigated already since the 1960s [11,12,13,14] as alloys for nuclear applications. The matrix/oxide interfaces are capable to absorb He atoms and the swelling due to He bubbles formation is significantly suppressed [14,15]. It is also well known that the attractive interaction between the dislocations and nanooxides leads to specific properties of the ODS alloys as threshold stress in high-temperature creep, under which the creep rate drops to immeasurable values [16,17,18]. Moreover, an appreciable strengthening of the ODS alloy can be achieved by nanosized oxide dispersion of a rather low volume fraction of 0.5%. This guarantees long-term shape stability of the parts loaded below the threshold, even at temperatures quite near the melting point of the matrix. This is, however, achieved only for stable coarse-grained ODS alloys, in which diffusional creep is suppressed. In this context, one should mention the works by Schneibel et al. [19,20,21] that demonstrate a fascinating resistance of ultra-fine-grained (UFG) ODS alloys against diffusional creep. However, according to [20] (see Figure 2 in this reference), the creep properties of coarse-grained ODS alloys surpass UFG ODS alloys for temperatures above 950 °C. Thus, one must insist on coarse-grained ODS alloys in applications of 1000–1300 °C.

As the ODS alloys cannot be produced by casting and subsequent heat treatment due to insufficient solubility of O in the liquid or solid alloy, the ODS alloys must be produced by applying the powder metallurgy consisting of the following steps.

1. During mechanical alloying (MA) of the input powders or granulates, the milling balls collide and small particles of powder attached to the ball surfaces are forged onto the balls to form growing protrusions. Large protrusions break off as large particles, which are again gradually crushed into small ones. In this way, the powder becomes more and more homogenized. In some studies, such as [22], it is shown that Y_2_O_3_ is only fragmented and homogeneously dispersed in the matrix during MA. However, more recent studies report that Y_2_O_3_ can completely be dissolved in the matrix during the late stages of MA and the O and Y atoms are trapped at drastically multiplied defects as dislocations and vacancies [23,24,25]. The degree of the chemical homogenization, as well as of the dissolution of Y_2_O_3_, depends critically on the time and conditions of MA. There exists a certain saturation limit, over which further MA does not contribute to the improvement of the final quality of the ODS alloy, and its properties can even worsen due to contamination from the abraded milling balls.
2. Canned evacuated ODS powder can be consolidated by spark plasma sintering [26], hot isostatic pressing [27], or hot forming as rolling [28], pressing [29], extrusion [30], rotary swaging [31,32], forging [33], or by their combinations [34,35]. Consolidation by hot forming leads usually to a UFG microstructure still containing a huge density of defects. Such an ODS alloy is hard and brittle and may contain sufficient stored energy to drive secondary recrystallization [36,37].
3. The secondary recrystallization takes place at high temperatures if the driving force, due to the energy of stored defects, overcomes the pinning of grain boundaries by the nanodispersoid. If the driving force is not too large, then only a small fraction of grains overcome the pinning effect and they grow to large sizes [38].

It is important to note that the ODS alloys keep a significant strength up to 1300 °C and the ‘crossing’ temperature with nickel-based superalloys is about 1100 °C. Unfortunately, the research dealing with properties and applicability of ODS alloys at temperatures of 1000–1300 °C is rather sporadic, according to [39,40].

The creep and oxidation-resistant Fe-10Al-4Cr-4Y_2_O_3_ (wt.% are used in the whole paper) ODS nanocomposite, denoted as the FeAlOY, has already demonstrated its promising potential for mechanically loaded long-term applications in the temperature range of 1100–1300 °C [31,36,38,41,42]. The FeAlOY is strengthened by 5 vol.% of nanodispersion of pure Y_2_O_3_ (the difference between 5 vol.% and 4 wt.% is given by the difference in densities of the matrix 6.5 g/cm^3^ and of Y_2_O_3_ 5.0 g/cm^3^), being the most resistant phase against coarsening [41,43], and has an appreciable resistance against oxidation up to 1400 °C [44].

For a successful introduction of the FeAlOY into practice, it is necessary to optimize all processing steps of its preparation. In this paper, we start with the optimization of MA as the process that consumes the most time and energy. MA is optimized under the condition to keep the final required microstructure and excellent high-temperature mechanical properties of the FeAlOY. That is why MA is performed at two different temperatures at different times. Then, the powder is analyzed with respect to its size distribution and chemical homogeneity. Moreover, microstructure and strength at 1100 °C and a strain rate of 10^−6^ s^−1^ of the final FeAlOY are characterized and the optimal conditions of MA are evaluated.

## 2. Materials and Methods

The authors have dealt with the development of the FeAlOY since 2014 [31,36,37,38,41,42,43,45,46]. The recent efforts include optimization of hot consolidation, secondary recrystallization, and chemical composition with respect to resultant microstructure and high-temperature mechanical properties. The processing steps of the FeAlOY preparation are described by following points.

(i) MA is performed in evacuated containers 400 mm in diameter and a volume of 22 dm^3^ filled with 100 bearing balls 40 mm in diameter (25 kg in total) rotating with 70 rpm along a horizontal axis. A total of 1.5 kg of FeAlOY powder is prepared in each container. The FeAlOY powder is produced from powders or granules of individual components with a purity of 99.9%.
(ii) After MA, the FeAlOY powder is canned in an evacuated consolidation steel container made from the 20/1 mm tube. The hot consolidation is carried out by rolling at a temperature of 900 °C in three steps to produce sheets of thickness at 3.25 mm of the fully dense FeAlOY.
(iii) After stripping the consolidation container, the FeAlOY is UFG, hard, and brittle at room temperature. To achieve the coarse-grained FeAlOY with excellent mechanical properties at temperatures over 1000 °C, the secondary recrystallization is provoked by annealing at a temperature over 1200 °C for 16 h. The recrystallized FeAlOY is relatively soft and ductile at room temperature and can be machined easily. The assertions concerning the mechanical properties of the UFG and the recrystallized FeAlOY are based on our results just prepared for publication.

To provoke precipitation of only the most stable pure Y_2_O_3_ nanodispersoid, O introduced into the system by oxidized powder surfaces is compensated by the addition of metallic Y granulates (about 1%). The addition leads also to a coarser grain microstructure after secondary recrystallization and a significant increase in the high-temperature strength of the FeAlOY [42]. The high Al content not only significantly improves the oxidation resistance but, surprisingly, also improves the high-temperature strength of the FeAlOY. Thus, the Al content is chosen as high as possible (10%) while still preserving the ferritic matrix. The addition of Cr supports the formation of larger grains after secondary recrystallization and also improves oxidation resistance [44,46,47].

Flat specimens with a gauge length of 25 mm and a cross-section of 2.5 mm × 3.5 mm are cut by a precise electrical discharge machine from the hot rolled and secondary recrystallized FeAlOY and ground to P400. Tensile tests are performed by using the Zwick/Roell KAPPA DS 50 kN creep test system (Zwick/Roell, Fürstenfeld, Austria) on air.

Microstructures are observed predominantly by using scanning electron microscope/microscopy (SEM) Tescan Lyra 3 XMU FEG/SEMxFIB (Tescan, Brno, Czech Republic) equipped with an X-Max80 EDS detector for X-ray microanalysis and a Symmetry 2 electron backscatter diffraction (EBSD) detector with an Aztec control system (Oxford Instruments, Abingdon, UK). The microstructure is also investigated by using a transmission electron microscope (TEM) JEOL 2100 F (JEOL Ltd., Akishima, Japan) at an acceleration voltage of 200 kV. The JEOL 2100F is operated in both TEM and STEM configurations with a Gatan Tridem GIF (EFTEM, EELS) (Gatan, Inc., Pleasanton, CA, USA) and an Oxford X-MaxN TSR EDS detector (Oxford Instruments plc., Abingdon, UK). An inverted optical microscope Olympus GX51 (Olympus Corporation, Shinjuku, Japan) is used for the characterization of the powder particle size. The metallographic cuts of the powders are analyzed using ImageJ software (version 1.53t) and the statistics are processed using MatLab 2022a software (Mathworks Inc., Natick, MA, USA).

The FeAlOY powders are prepared according to the procedure described in item (i). MA is performed simultaneously in four containers and two of them are air-cooled by a fan. The temperature in the room is kept at 25 °C, the surfaces of air-cooled containers are kept at 33 °C, and the surface temperature of the remaining two containers is kept at 46 °C. The temperature is measured by a radiation thermometer and checked by a contact thermometer.

To analyze the FeAlOY powder after MA, the powder is mixed into the resin. After hardening the resin tablet, its metallographic cut is performed by grinding up to 2500 SiC paper, mechanically polished (3 μm and 1 μm diamond paste, 5 min each step), and finished with oxide polishing suspension (OPS). The powder is analyzed by optical microscopy and in SEM by EDS and back-scattered electrons (BSE). The EBSD mapping of the recrystallized FeAlOY is also performed on metallographic cuts prepared by grinding up to 2500 SiC paper, mechanical polishing (3 μm and 1 μm diamond paste, 5 min each step), and finished in VibroMet (MasterMet2 suspension, 400 g weights, 3 h). Moreover, the nanooxide dispersion in the FeAlOY is analyzed in SEM by secondary electrons (SE) on a metallographic cut etched with a Villela–Bain etchant (2 g picric acid, 100 mL ethanol, 5 mL hydrochloric acid) for 5 s.

## 3. Results and Discussion

The times of MA of the FeAlOY powder at temperatures of 33 and 46 °C are chosen as 60, 90, 120, 170, 350, and 500 h. The batches are denoted as *C* (33 °C) and *W* (46 °C) followed by the time of the MA, e.g., *C*90 or *W*170.

### 3.1. Analysis of the FeAlOY Powders after Different Times of MA

**Figure 1:**
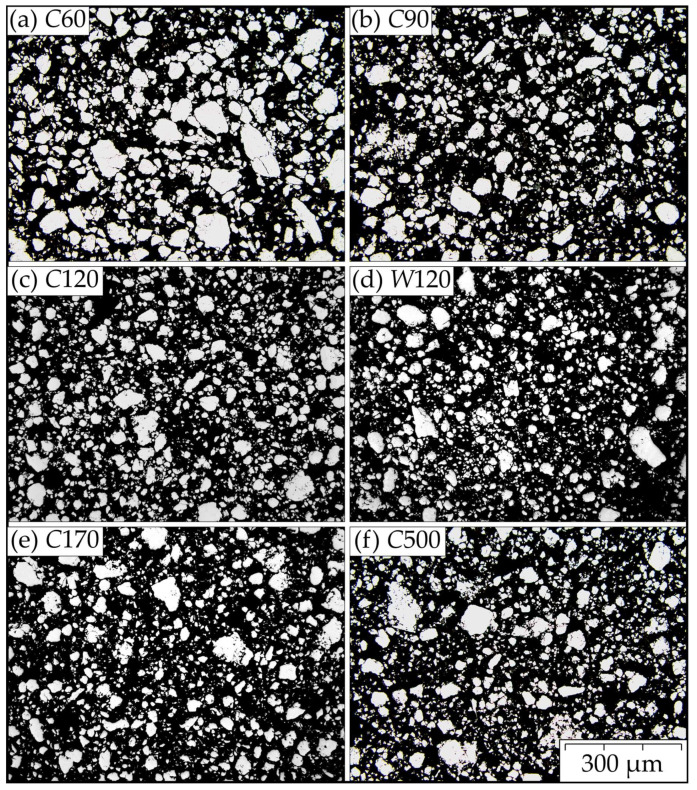
Representative metallographic cuts of powders (**a**) *C*60, (**b**) *C*90, (**c**) *C*120, (**d**) *W*120, (**e**) *C*170, and (**f**) *C*500 (optical microscope, the contrast is adjusted for better distinguishing between the powder particles (white) and the resin (black)).

**Figure 2:**
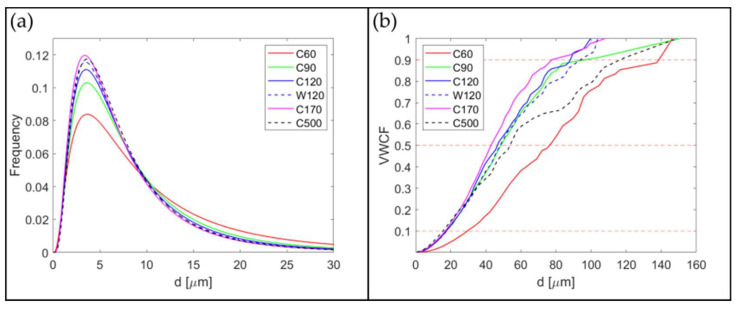
Distribution functions representing (**a**) log-normal size distribution of powder particles detected at the metallographic cut and (**b**) Volume Weighted Cumulative Frequency (VWCF) of powder particle sizes.

Metallographic cuts of the powders after MA *C*60, *C*90, *C*120, *W*120, *C*170, and *C*500 are prepared and evaluated. Representative metallographic cuts demonstrating the rather wide particle size distributions are presented in Figure 1. All metallographic cuts are evaluated by an automatic image analyzer providing the area $\bar{A}$ distributions at the particle cuts. By assuming that the particle is spherical and cut randomly, one can estimate the value of the area A of the particle cut across its equator as $A=\bar{A}/\int_{0}^{1}\left(1-x^{2}\right)\mathrm{dx}=3\bar{A}/2$. Then, the diameter of the particle (particle size) is given by $d=\sqrt{4A/\pi }$. The frequency F of the particles in the metallographic cut corresponding to the size d is presented in Figure 2a. The data are smoothed by fitting to the log-normal distribution function. To imitate the sieve analysis of the powders, one should take into consideration that the probability of cutting the particle is proportional to its size d. The standard outcome of the sieve analysis is the cumulative passing mass fraction representing the weight fraction of the powder particles with a size less than d. Let us denote this function as Volume Weighted Cumulative Frequency (VWCF). As the mass of the particle is proportional to $d^{3}$ and the probability of its cutting is proportional to d (the number of large particles is overestimated in the cut), the VWCF function can be calculated as integral from 0 to d of the product of F and $d^{2}$ (see Figure 2b). Each distribution function is evaluated from five micrographs similar to those shown in Figure 1. The results in Figure 2a clearly indicate some influence of the time of MA on the shape of the size distribution of the function F, but only for times of MA up to 120 h. In Figure 2b, one can conclude that the particles of size $d=\left(40\pm 10\right)\;\mu m$ have the highest proportion by weight in the powder for times of MA over 90 h. The representative statistics for the powder particle sizes are shown in Table 1. For effective cold compaction and hot consolidation of the powders, a wide-size distribution function is welcome, as small particles can effectively fill up the space between the larger ones. Judging only by the particle size distributions, the *C*60 and *C*500 powders have the most favorable size distributions, while *C*120 and *W*120 are the least favorable. However, the differences are not very pronounced.

**Figure 3:**
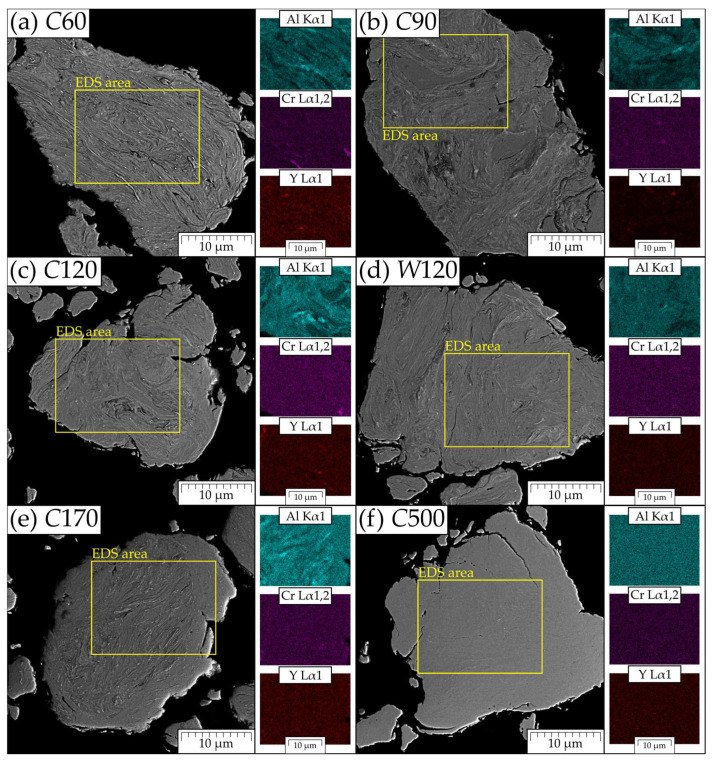
BSE images and corresponding EDS mapping of powder particles of (**a**) *C*60, (**b**) *C*90, (**c**) *C*120, (**d**) *W*120, (**e**) *C*170, and (**f**) *C*500.

**Figure 4:**
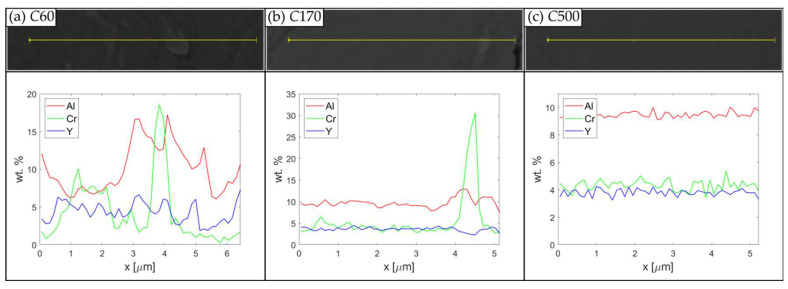
EDS line scans of powder particles of (**a**) *C*60, (**b**) *C*170, and (**c**) *C*500.

The same metallographic cuts are also used for the SEM analyses of the chemical inhomogeneities by BSE and EDS mapping (Figure 3). From the figure, one can see, that increasing the time of MA leads systematically to the homogenization of Al, Cr, and Y elements and an ideal homogenization is achieved in the case of *C*500. Rather significant inhomogeneities are clearly visible in cases of shorted times of MA (*C*60 and *C*90). Note also that *W*120 is significantly more homogenous than *C*120. Shorter time and lower temperature of MA lead not only to poor chemical homogenization predominantly of Al and Cr, but also to incomplete dissolution and insufficient dispersion of the Y_2_O_3_ particles. To obtain a quantitative image of the chemical inhomogeneities and their evolution in the time of MA, EDS line scans are performed for *C*60, *C*170, and *C*500 and shown in Figure 4. In the figure one can see that the inhomogeneity of *C*60 is significant in all components; in *C*170 one can find some small Cr particles as residuum of input Cr granules and *C*500 can be considered homogeneous in all components. It is, however, worth noting that certain chemical inhomogeneities of metallic components, such as Al and Cr, can be tolerated, as annealing the consolidated specimens to provoke their secondary recrystallization leads to chemical homogenization of the matrix by diffusion.

**Figure 5:**
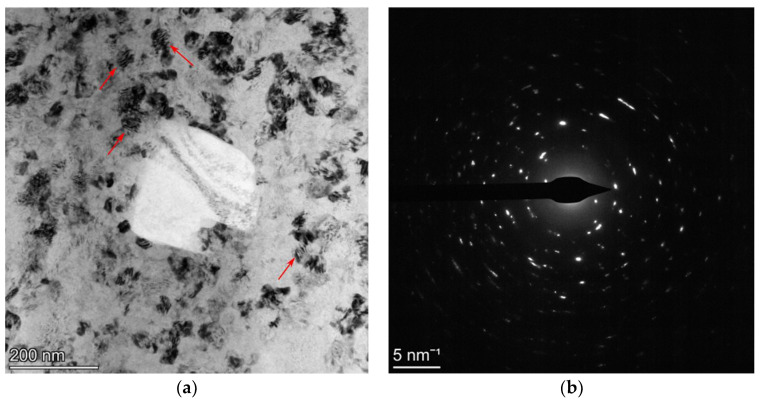
TEM micrograph of the powder *C*60 (**a**) showing the UFG microstructure a size of about 50 nm with a very high dislocation density of the order of magnitude of 10^16^ m/m^3^ (estimated from the distances between dislocations; about 10 nm detected in the grains are pointed by arrows) and a rather exceptional large undissolved Y_2_O_3_ particle, and (**b**) showing the selected area electron diffraction pattern of the UFG microstructure and proving the developed crystal structure of the grains.

The attempts to reveal the grain microstructure in the powder particles by using BSE or EBSD mapping at the metallographic cuts are not successful due to the very small grain size and very high dislocation density. That is why the powder *C*60 is analyzed by TEM, showing the UFG microstructure of the FeAlOY powder of about 50 nm (Figure 5). Moreover, one can also estimate a rather high density of dislocations in the grains and not fully developed (unsharp) boundaries between grains rather represented by zones with concentrated dislocations. The dislocation density of the order of magnitude 10^16^ m/m^3^ can be estimated, which corresponds to the distances between dislocations (about 10 nm) detected in the grains pointed by arrows. We assume that very similar results will be also obtained for other batches.

### 3.2. Tensile Tests of the Recrystallized FeAlOY Prepared from Powders after Different Times and Temperatures of MA

**Figure 6:**
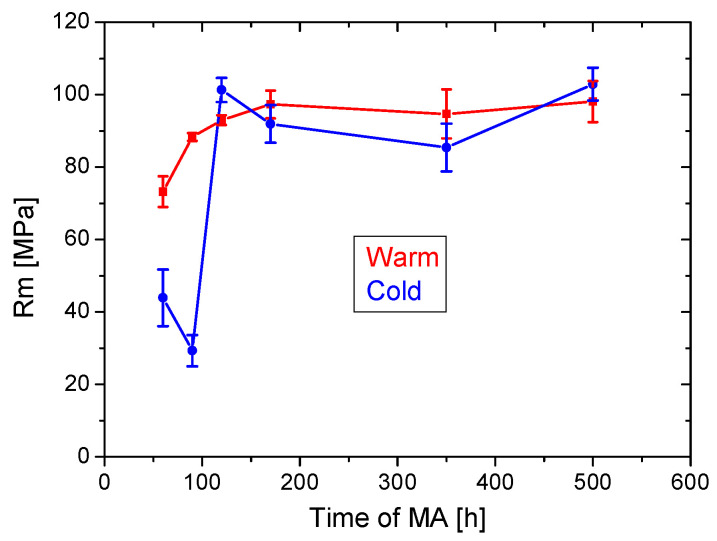
Dependence of average ultimate strength *Rm* on the time of MA.

The tensile tests of the FeAlOY are performed at 1100 °C and the strain rate is 10^−6^ s^−1^, resembling creep conditions. Four specimens are used for each batch corresponding to the given conditions of MA (the time and temperature of MA). The average ultimate strengths *Rm* with error bars is depicted in Figure 6. The results clearly indicate that the strength of the FeAlOY becomes saturated for MA after about 150 h at both temperatures. For shorter times of MA, the strength significantly decreases and the tensile tests indicate that MA at 33 °C is less effective and reliable than at 46 °C. To explain the effects, the microstructure of the secondary recrystallized specimens is also analyzed.

### 3.3. Microstructure Analysis of the Recrystallized FeAlOY Prepared from Powders after Different Times and Temperatures of MA

Two microstructural features are analyzed in the recrystallized FeAlOY: the influence of MA parameters on the grain microstructure and the morphology of the nanooxide dispersoid.

**Figure 7:**
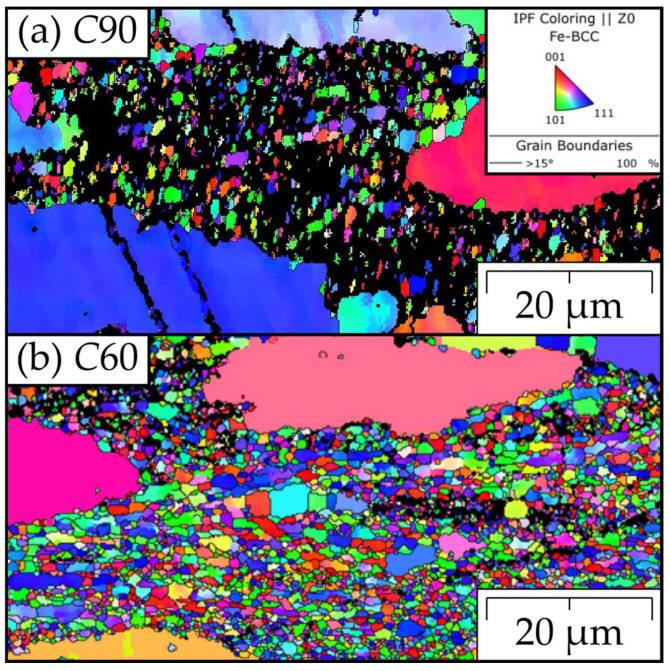
Representative IPF-Z EBSD maps of the unfavorable grain microstructures of (**a**) *C*90 and (**b**) *C*60. The grain microstructure in (**a**) is not fully recrystallized with a large amount of non-indexed regions, where the grains are either too small or the extreme dislocation density prevents the formation of pronounced Kikuchi patterns. The grain microstructure in (**b**) is better developed than in (**a**); however, the grains are small and thus unfavorable towards high-temperature strength.

**Figure 8:**
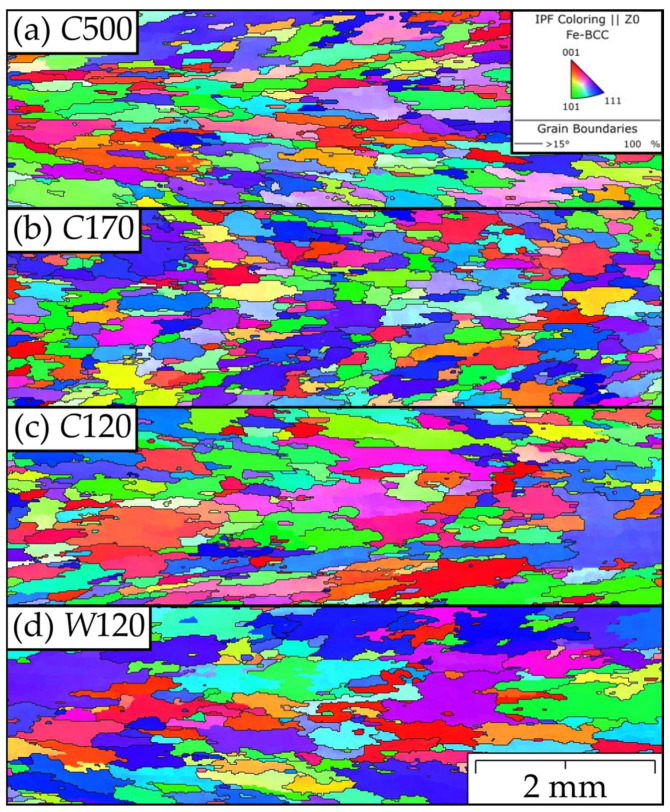
Representative IPF-Z EBSD maps of the favorable coarse-grained microstructures (**a**) *C*500, (**b**) *C*170, (**c**) *C*120, and (**d**) *W*120. These specimens are fully recrystallized and elongated in the direction of loading (horizontal direction on the maps).

The representative EBSD maps of the grain microstructures are shown in Figure 7 and Figure 8, clearly indicating that the drop in the strength of the *C*60 and *C*90 specimens of the FeAlOY is due to incomplete recrystallization. The fine-grained regions shown in Figure 7 are much softer at 1100 °C, causing significant stress concentrations during the tensile test, which leads to a drop in strength. All other batches are completely recrystallized (see Figure 8 for examples), which is exhibited by the much higher strength. The grain size distributions of fully recrystallized specimens are evaluated in Table 2.

**Figure 9:**
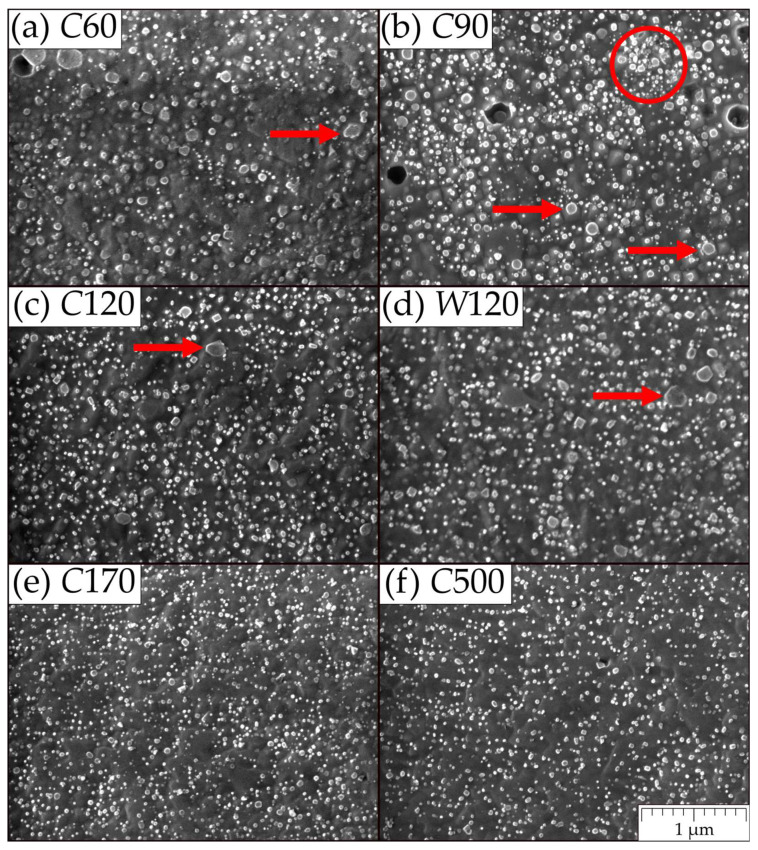
Representative morphologies of the nanooxide dispersoid of (**a**) *C*60, (**b**) *C*90, (**c**) *C*120, (**d**) *W*120, (**e**) *C*170, and (**f**) *C*500. Red arrows point out larger oxide particles and the red circle in (**b**) shows unfavorable clustering of smaller oxides.

The representative micrographs of the morphology of the nanooxide dispersoid are shown in Figure 9. The nanooxide dispersoid in *C*60 and *C*90 specimens is less favorable, as some of the oxide particles are large or clustered, while the examples of more favorable *C*170 and, especially, *C*500 show fine homogeneously distributed oxide nanoparticles. In Figure 9, one can deduce that the large oxides of 50–200 nm in size indicated by arrows in Figure 9a–d (the amount of these oxides decrease with the time of MA) stem from undissolved Y_2_O_3_ during MA. Small oxides are shown in Figure 9 precipitate during hot consolidation to sizes of about 5 nm [38] and coarsen to sizes of 20–30 nm during secondary recrystallization. By comparison, in Figure 9e,f, one can conclude that the time of 170 h is sufficient for a nearly complete dissolution of Y_2_O_3_ during MA. It should be noted that the coarsening of oxides in the UFG microstructure is significantly accelerated due to fast grain boundary diffusion [38] and it becomes much slower in coarse grains [41]. Furthermore, the oxides shown in Figure 9 are denuded by etching, the time of which significantly influences the area density of oxides. Moreover, one can also observe rather different area fractions of oxides in different grains due to different etching velocities of differently oriented grains. Thus, the volume fraction cannot be estimated from Figure 9 and its most reliable value can be deduced from the chemical composition of the FeAlOY.

### 3.4. Relations between Parameters of Powders, Recrystallized Microstructures, and Tensile Strength

One of the goals of the present paper is to elucidate the influence of the conditions of MA on the microstructure of the powders and the microstructure and high-temperature strength of the secondary recrystallized FeAlOY. The results clearly indicate that the increase in the time of MA leads to improvement of the FeAlOY only up to a certain limit. Moreover, the results indicate that MA at higher temperatures of 46 °C are more effective than at 33 °C.

If one compares the results of the microstructure of the powders with the microstructure of dispersion of the recrystallized FeAlOY, the results are plausible. Longer time and higher temperature of MA lead to better homogenization of the powder interiors, resulting in a finer and more homogeneous dispersion in the recrystallized FeAlOY. However, the difference between *C*170 and *C*500 is rather insignificant so the increase in the time of MA from 170 h to 500 h is not reasoned. The same conclusion can also be performed when evaluating the influence of the microstructure in the powder interiors on the grain microstructure of the recrystallized FeAlOY. Here, the influence is significant only for batches *C*60 and *C*90, where the insufficient homogeneity of the powders leads to incomplete secondary recrystallization of the FeAlOY.

If one evaluates the relationship between the microstructure of the recrystallized FeAlOY and its strength, the most significant feature is the drastic drop in the strength at 1100 °C caused by incomplete recrystallization. This sends a clear signal that incompletely recrystallized microstructures of the ODS alloys are very detrimental to their high-temperature strength. The decrease in the strength of *W*60 compared to other *W* batches can be attributed to inhomogeneities in the dispersoid. The strength of all other batches is on a similar level. One can thus conclude that the mechanical alloying at 46 °C for 170 h is the optimized condition for MA, guaranteeing expected microstructure and excellent high-temperature properties of the FeAlOY.

## 4. Conclusions

The main goal of the present paper is to evaluate the optimal conditions for MA under the condition of keeping the required microstructure and excellent high-temperature mechanical properties of the recrystallized FeAlOY. The conclusions can be summarized in the following items.

- The time and temperature of MA do not significantly influence the rather wide size distribution of the FeAlOY powders. Only for the shortest times of MA is the fraction of small-sized particles slightly lower and the fraction of very large particles is increased in comparison to other conditions of MA.
- Increasing the time and temperature of MA leads to better and better homogenization of the powder particle interiors. A significant dispersion and dissolution of Y_2_O_3_ powders are achieved at the shortest times of MA, while the chemical homogeneity of the metallic components is achieved only for the longest times of MA. The inhomogeneity of the metallic components is, however, tolerable, as sufficient homogeneity in the FeAlOY is achieved by diffusion during its annealing to provoke its secondary recrystallization.
- MA at 46 °C provides better and more reliable results than MA at 33 °C.
- Times of MA that are too short lead to inhomogeneous oxide dispersion in the FeAlOY and may lead to its incomplete secondary recrystallization with the consequence of a drastic drop in its mechanical properties at high temperatures.
- The optimal conditions of MA in our attritor are a temperature of 46 °C (without cooling by air blower) and a time of 170 h (one week).

## Figures and Tables

**Table 1:** Characterization of powder particle sizes distribution calculated from log-normal size distribution (see Figure 2a) and volume-weighted sieving parameters $d_{X\%}$ for 10, 50, and 90% values determined from volume-weighted cumulative frequency (see Figure 2b).

Conditions of MA	Time of MA	d	$d_{10\%}$	$d_{50\%}$	$d_{90\%}$
Cold/Warm	[h]	[μm]	[μm]	[μm]	[μm]
*C*	60	11.7 ± 12.7	29.4	76.3	138.5
*C*	90	9.1 ± 8.4	17.4	48.6	96.5
*C*	120	8.4 ± 7.4	17.6	46.6	88.1
*W*	120	8.1 ± 7.0	17.6	48.2	93.8
*C*	170	7.8 ± 6.7	16.9	43	77.7
*C*	500	7.9 ± 6.5	15.6	53.7	116.6

**Table 2:** Grain size analysis of consolidated recrystallized powders. The batches *C*60 and *C*90 are not presented, as the recrystallization is incomplete in the specimens.

Conditions of MA	Time of MA	Mean Area	Area-Weighted Mean Area
Cold/Warm	[h]	[μm^2^]	[μm^2^]
*C*	500	55,094.4	291,253.0
*C*	170	38,949.9	97,183.5
*C*	120	57,801.7	303,532.1
*W*	120	83,156.5	412,319.5

## Data Availability

Available upon request.
